# Two-Year Outcomes after Left Main Coronary Artery Percutaneous Coronary Intervention in Patients Presenting with Acute Coronary Syndrome

**DOI:** 10.1155/2020/6980324

**Published:** 2020-04-06

**Authors:** Si-Da Jia, Yi Yao, Ying Song, Xiao-Fang Tang, Xue-Yan Zhao, Run-Lin Gao, Yue-Jin Yang, Bo Xu, Zhan Gao, Jin-Qing Yuan

**Affiliations:** Fu Wai Hospital, National Center for Cardiovascular Diseases, Peking Union Medical College and Chinese Academy of Medical Sciences, Beijing, China

## Abstract

**Objectives:**

We aim to evaluate long-term outcomes after left main coronary artery (LMCA) percutaneous coronary intervention (PCI) in patients presenting with acute coronary syndrome (ACS).

**Background:**

PCI of the LMCA has been an acceptable revascularization strategy in stable coronary artery disease. However, limited studies on long-term clinical outcomes of LMCA PCI in ACS patients are available.

**Methods:**

A total of 6429 consecutive patients with ACS undergoing PCI in Fuwai Hospital in 2013 were enrolled. Patients are divided into LMCA group and Non-LMCA group according to whether the target lesion was located in LMCA. Prognosis impact on 2-year major adverse cardiovascular and cerebrovascular events (MACCE) is analyzed.

**Results:**

155 (2.4%) patients had target lesion in LMCA, while 6274 (97.6%) patients belong to the non-LMCA group. Compared with non-LMCA patients, LMCA patients have generally more comorbidities and worse baseline conditions. Two-year follow-up reveals that LMCA patients have significantly higher rate of cardiac death (2.6% vs. 0.7%, *p* = 0.034), myocardial infarction (7.1% vs. 1.8%, *p* < 0.001), in-stent thrombosis (4.5% vs. 0.8%, *p* < 0.001), and stroke (7.1% vs. 6.4%, *p* = 0.025). After adjusting for confounding factors, LMCA remains independently associated with higher 2-year myocardial infarction rate (HR = 2.585, 95% CI = 1.243–5.347, *p* = 0.011).

**Conclusion:**

LMCA-targeted PCI is an independent risk factor for 2-year myocardial infarction in ACS patients.

## 1. Introduction

For patients with low SYNergy between percutaneous coronary intervention with TAXus and cardiac surgery (SYNTAX) score, percutaneous coronary intervention (PCI) has been recommended as a reasonable revascularization strategy in patients with significant stenosis in the left main coronary artery (LMCA) presenting with stable coronary artery disease (SCAD) [1]. Previous studies have proven that, compared with coronary artery bypass graft (CABG) surgeries, unprotected LMCA-targeted PCI resulted in similar rate of mortality and composite event of death, myocardial infarction (MI), and stroke [2–10]. However, studies on long-term clinical outcomes of LMCA PCI in acute coronary syndrome (ACS) patients are relatively rare.

A limited number of studies have yielded conflicting results in terms of LMCA PCI in ACS settings. Several studies found that, although patients with AMI and thrombosis in unprotected LMCA are at high-risk for substantial mortality, PCI is still associated with a remarkably high short-term and long-term survival rates [11–13]. Moreover, in another study reported by Gao et al. [14], transradial PCI on unprotected LMCA and/or multivessel disease for patients with ACS had comparable clinical outcomes to CABG, with an advantage of reducing stroke. Contrarily, Baek et al. [15] found patients with ST elevation myocardial infarction (STEMI) and LMCA PCI had poor clinical outcome, which is attributable to periprocedural hemodynamic deterioration. A recent analysis from the EXCEL trial has found that patients with LMCA disease undergoing PCI or CABG had similar rate of adverse events irrespective of the acuity of clinical presentation [16]. However, these studies were modest in sample size or failed to observe the long-term outcome of LMCA PCI in real-world clinical settings.

Thus, we aim to evaluate long-term clinical outcome of LMCA PCI in patients presenting with ACS in our real-world, prospective, large-sample cohort of Chinese patients.

## 2. Materials and Methods

### 2.1. Study Population

Data from all consecutive patients from a single center (Fu Wai Hospital, National Center for Cardiovascular Diseases, Beijing, China) undergoing PCI were prospectively collected. Based on contemporary practice guidelines, revascularization strategies were finally determined by heart team discussion involving interventional cardiologists, cardiac surgeons, and physicians. Patients who did not undergo PCI and were referred for CABG after heart team discussion were excluded from this study. Between January 2013 and December 2013, a total of 10,724 consecutive patients were enrolled undergoing PCI. The Institutional Review Board approved the study protocol, and the patients provided written informed consent before the intervention.

Exclusion criteria included patients presenting with SCAD (*n* = 4,295). Thus, 6,429 ACS patients undergoing PCI were included in the present study. Patients presenting with ACS and LMCA stenosis requiring percutaneous coronary intervention were included in the LMCA group, while other patients enrolled in this study were stratified into the non-LMCA group.

### 2.2. Procedure and Medications

The PCI strategy and stent type were left to treating physician's discretion. ACS patients (STEMI and NSTE-ACS) scheduled for PCI received the same dose aspirin and ticagrelor or clopidogrel (loading-dose 300 mg or 600 mg) as soon as possible. During the procedure, unfractionated heparin (100 U/kg) was administered to all patients, and use of glycoprotein IIb/IIIa inhibitors was per operator's judgment. After the procedure, aspirin was prescribed at a dose of 100 mg daily indefinitely; clopidogrel 75 mg daily or ticagrelor 90 mg twice daily was advised for at least 1 year after PCI.

### 2.3. Patient Follow-Up

All patients were evaluated by clinic visit or by phone at 1, 6, and 12 months and annually thereafter. Patients were advised to return for coronary angiography if clinically indicated by symptoms or documentation of myocardial ischemia.

### 2.4. Endpoints and Definitions

Death resulting from any reason, including cardiac death, was defined as all-cause death. Death that could not be attributed to a noncardiac etiology was considered cardiac death. MI was defined by the third universal definition of MI [17]. Revascularization was defined as repeated revascularization for ischemic symptoms and events driven by PCI or surgery of any vessel. Unplanned target vessel revascularization (TVR) was defined as repeat percutaneous intervention or surgical bypass of any segment of the target vessel for ischemic symptoms and events driven [18]. Stent thrombosis (ST) was defined according to the Academic Research Consortium, including definite, probable, and possible in the analysis [18]. Bleeding was quantified according to Bleeding Academic Research Consortium (BARC) definition criteria, including types 2, 3, and 5 in the analysis [19]. Major adverse cardiac and cerebrovascular event (MACCE) was defined as the occurrence of death, MI, TVR, ST, and stroke during follow-up. All endpoints were adjudicated centrally by 2 independent cardiologists, and disagreement was resolved by consensus. The SYNTAX score was assessed by two of three experienced cardiologists in an independent angiographic core lab and were blinded to the clinical outcomes. In case of disagreement among the two observers, opinion from the third cardiologist was obtained to finally reach a consensus.

### 2.5. Statistical Analysis

Continuous variables are expressed as mean ± standard deviation, and categorical variables are presented as percentages. Differences in baseline characteristics and in-hospital outcomes between groups were assessed using the chi-squared test or Fisher's exact test for categorical variables and Student's *t*-test or the Wilcoxon rank test for continuous variables, as appropriate. Survival curves were constructed using Kaplan–Meier method, and the log-rank test was performed to compare the time to clinical endpoints. Cox regression analyses were conducted to evaluate the adjusted effect of LMCA PCI on 2-year clinical endpoints. Clinically and statistically significant covariates were all entered into the model, and results were reported as adjusted hazard ratios together with corresponding 95% confidence intervals (CI). Adjusted confounding factors include LMCA, age, diabetes, hemoglobin, GFR, STEMI, UA, preprocedural SYNTAX score, puncture site, staged PCI, IVUS, IABP, successful PCI, and stent type. For all analyses, a 2-sided *p* value < 0.05 was considered significant. Statistical analysis was performed using IBM® SPSS® v22.0.0.0 software (SPSS Inc., Chicago, IL, USA).

To minimize the effect of confounding factors caused by differences in baseline characteristics between LMCA and non-LMCA groups, and propensity score match (PSM) was performed. A propensity score was estimated for each patient using a logistic regression model. Patients were matched on estimated propensity scores, using a nearest neighbor approach. The matched variables were LMCA, age, diabetes, hemoglobin, GFR, STEMI, UA, preprocedural SYNTAX score, puncture site, staged PCI, IVUS, IABP, successful PCI, and stent type.

## 3. Results

Among 6,429 patients presenting with ACS undergoing PCI, 155 (2.4%) patients was in the LMCA group, while 6,274 (97.4%) patients belonged to the non-LMCA group. Median follow-up time was 760 days. 147 (94.8%) of patients in the LMCA group had unprotected LMCA. Before PSM, compared with the non-LMCA group, patients in the LMCA group are older, with higher proportion of diabetes, more clinical presentation of unstable angina (UA), and *β*-blocker usage. Laboratory findings indicated lower levels of hemoglobin and glomerular filtration rate (GFR) for LMCA patients (Table 1). In terms of angiographic and procedural findings, LMCA patients are associated with higher preprocedural SYNTAX score, higher rate of trivessel disease, staged PCI, usage of intravascular ultrasound (IVUS) and intraaortic balloon pump (IABP) support, and fewer implantations of first-generation drug-eluting stents (1G-DES) (Table 2). After PSM, all matched baseline characteristics were no longer significantly different, despite introducing a few new imbalance in baseline and angiographic characteristics between groups.

**Figure 1:**
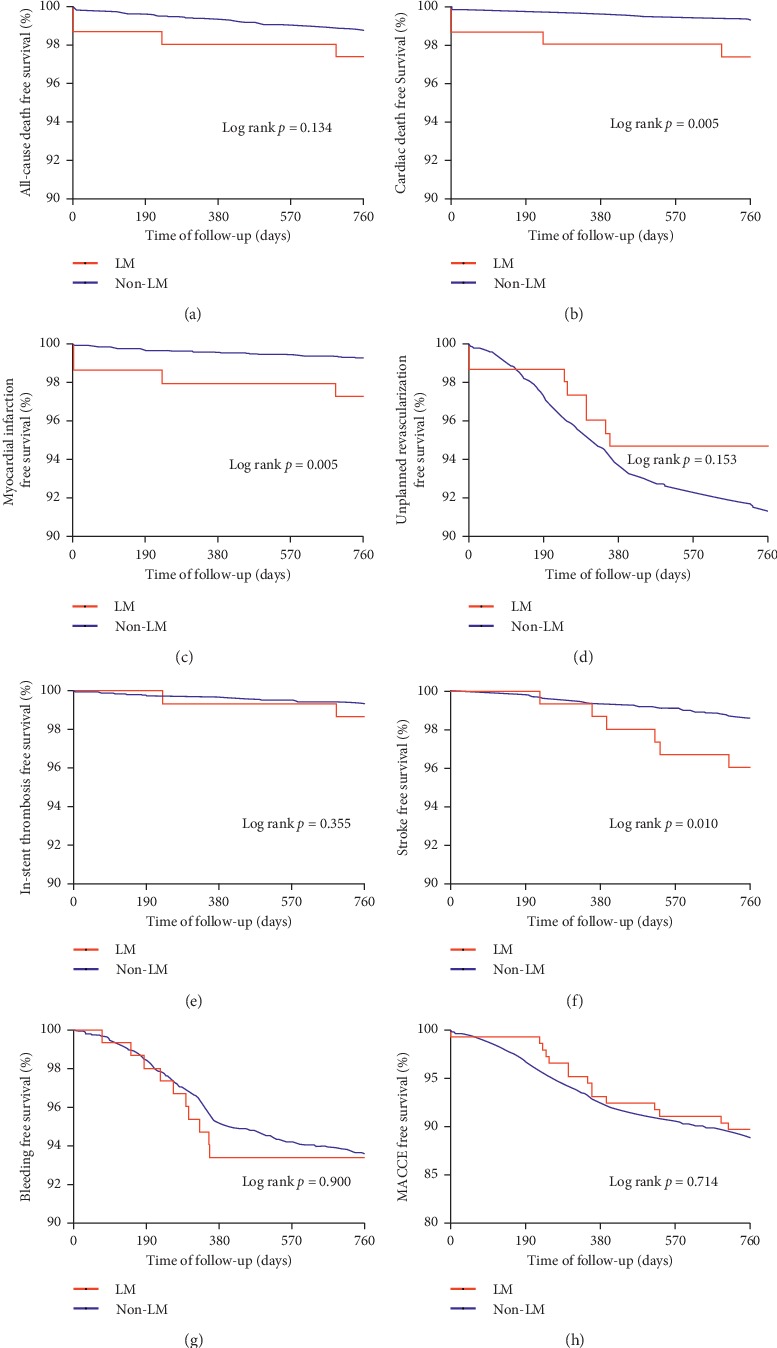
Kaplan–Meier survival curves on 2-year clinical endpoints between LMCA and non-LMCA groups for patients presenting with ACS. (a) All-cause death; (b) cardiac death; (c) myocardial infarction; (d) unplanned revascularization; (e) in-stent thrombosis; (f) stroke; (g) bleeding; (h) MACCE. MACCE = major adverse cardiac and cerebrovascular events.

Before PSM, follow-up results revealed that LMCA patients had higher incidence of 2-year cardiac death (2.6% vs. 0.7%, *p*=0.034), target vessel MI (5.2% vs. 0.8%, *p*=0.001), in-stent thrombosis (4.5% vs. 0.8%, *p*=0.001), and stroke (3.9% vs. 1.4%, *p*=0.025), while no difference was observed for all-cause death, target lesion MI, unplanned revascularization, bleeding, and MACCE (all *p* > 0.05) (Table 3). Kaplan–Meier survival analysis revealed similar findings, except that no difference was observed for 2-year in-stent thrombosis-free survival (Figure 1). After PSM, the incidence of most clinical outcomes was nonsignificant between LMCA and non-LMCA groups, except for a significantly higher rate of MI in the LMCA group (3.0% vs. 1.3%, *p*=0.011).

**Figure 2:**
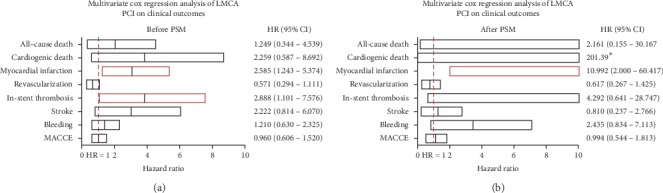
Adjusted hazard ratios of LMCA PCI for clinical outcomes. HR and 95% CI of each clinical outcome are shown in the plot. Clinical outcomes shown in red bars are significantly different. (a) Before propensity score match; (b) after propensity score match. MACCE = major adverse cardiac and cerebrovascular event. Adjusted variables: LMCA, age, diabetes, hemoglobin, GFR, STEMI, UA, preprocedural SYNTAX score, puncture site, staged PCI, IVUS, IABP, successful PCI, and stent type. ^*∗*^Data unavailable as no patient had cardiac death in the non-LMCA group after PSM.

Before PSM and after adjusting for differences by Cox regression analysis in age, diabetes, hemoglobin and GFR levels, clinical presentation, preprocedural SYNTAX score, puncture site, staged PCI, IVUS and IABP usage, successful PCI, and stent type, we found LMCA was independently associated with higher risk of 2-year MI (HR = 2.585, 95% CI = 1.243–5.347, *p*=0.011) and in-stent thrombosis (HR = 2.888, 95% CI = 1.101–7.576, *p*=0.031). For all-cause death, cardiogenic death, unplanned revascularization, stroke, bleeding, and MACCE, the difference was nonsignificant (all *p* > 0.05). After PSM, however, only difference in occurrence of 2-year MI (HR = 10.992, 95% CI: 2.000–60.417, *p*=0.006) remained statistically significant, while difference in 2-year in-stent thrombosis rate became nonsignificant (Figure 2).

Subgroup analysis revealed that LMCA patients presenting with STEMI had higher risk of 2-year all-cause death (12.5% vs. 3.0%, *p*=0.013), cardiac death (12.5% vs. 1.3%, *p*=0.005), MI (16.7% vs. 2.2%, *p*=0.002), and in-stent thrombosis (12.5% vs. 1.0%, *p*=0.002) compared with non-LMCA patients, while no difference was found between LMCA and non-LMCA in unplanned revascularization, stroke, bleeding, and MACCE (All *p* > 0.05). Compared with non-LMCA patients, LMCA patients presenting with UA/non-ST elevation myocardial infarction (NSTEMI) had higher incidence of MI (5.3% vs. 1.8%, *p*=0.010), in-stent thrombosis (3.1% vs. 0.8%, *p*=0.022), and stroke (4.6% vs. 1.5%, *p*=0.016), while no difference was observed in all-cause death, cardiac death, unplanned revascularization, bleeding, and MACCE (All *p* > 0.05) (Table 4).

## 4. Discussion

In our study, patients presenting with ACS undergoing LMCA PCI was compared with ACS patients undergoing non-LMCA PCI in a large cohort of Chinese patients undergoing contemporary PCI. The main finding of this study is as follows: (1) in patients presenting with ACS, LMCA-targeted PCI is associated with higher risk of 2-year cardiac death, MI, in-stent thrombosis, and stroke. (2) Compared with non-LMCA-targeted PCI, LMCA-targeted PCI is an independent risk factor for 2-year MI.

For SCAD patients with LMCA disease undergoing revascularization, PCI is recommended in the 2018 ESC/EACTS Guidelines on Myocardial Revascularization (Class I for low SYNTAX score and class IIa for intermediate SYNTAX score) [1]. In recent years, a number of randomized controlled studies have compared the long-term clinical outcomes of patients LMCA disease undergoing different revascularization procedures. The EXCEL [10] and PRECOMBAT [20] study showed similar rate of long-term adverse events between patients undergoing LMCA PCI or CABG, while NOBLE trial [21] findings suggested that CABG might still be a better option for these patients. However, little is known for the long-term effect of PCI in ACS patients with significant LMCA disease. Sim et al. found acute MI patients with a culprit LMCA having higher in-hospital mortality than patients with nonculprit LMCA, while 1-year clinical outcomes were similar [22]. In a larger observational study [23], long-term survival rates (median follow-up time was 6.3 years) were similar between STEMI patients and UA/NSTEMI patients due to unprotected LMCA disease. The DELTA all-comer, a multinational registry-revealed PCI for ACS in ULMCA is associated with similar rate death, cerebrovascular accident, and MI compared with CABG at long-term follow-up [24]. Patel et al. [25] found unprotected LMCA occlusion in patients undergoing primary PCI is independent predictor of 30-day and 3-year all-cause mortality. To the best of our knowledge, there is no large-sample observational study available yet to compare long-term clinical outcomes of ACS patients undergoing PCI between LMCA and non-LMCA as the target vessel.

ACS patients with significant unprotected LMCA disease represent one of the most high-risk types of coronary artery disease, especially for patients with acute MI caused by LMCA culprit lesion. As LMCA supplies blood perfusion for the majority of left ventricle myocardium regardless of coronary artery dominance, acute LMCA infarction leads to large infarction area [26]. A large proportion of AMI patients with LMCA involvement present cardiogenic shock, who are at much higher risk of in-hospital and short-term mortality [27]. Unfortunately, cardiogenic shock data are unavailable in our cohort database, so this serious complication was not further analyzed in this study. It is worth noticing that in the LMCA group, the vast majority of patients had unprotected LMCA (94.8%). With a patent bypass graft to distal coronary arteries, it is reasonable to infer ACS patients with protected LMCA have better prognosis compared with patients with unprotected LMCA. Thus, high proportion of unprotected LMCA in the LMCA group also contributed to worse clinical outcomes. Furthermore, our patients with significant LMCA disease are complicated by other characteristics and comorbidities leading to higher mortality, including higher age, lower GFR level, and more patients with diabetes. Finally, patients in the LMCA group had generally more complex coronary lesions compared with non-LMCA patients, as characterized by higher proportion of trivessel disease and higher level of preprocedural SYNTAX score. These factors all contribute to higher cardiac mortality in LMCA group in our study population, despite efforts to improve patient survival by less use of first-generation DES, more use of IABP support, IVUS guidance, and staged PCI to treat multivessel disease in the LMCA group. After adjustment of baseline characteristics that are significantly different across the groups using Cox regression analysis, however, the incidence of cardiac death and stroke between the groups was no longer significantly different, indicating the higher risk of 2-year cardiac mortality and stroke in the LMCA group can be attributed to patients' poorer general baseline condition.

Clinical presentation in our patient population is an important indicator for acute and long-term prognosis. Compared with less severe conditions like UA or NSTEMI, patients presenting with STEMI with LMCA culprit lesion are critically ill, often leading to abrupt circulatory failure, fatal ventricular arrhythmia, and sudden cardiac death [28]. Since STEMI with culprit LMCA often leads to sudden death before the patient reaches hospital, we see a significantly lower proportion of hospitalized STEMI patients in the LMCA group (15.5% vs. 22.6%, *p*=0.035), which is in accordance with a previous report [13]. Due to the heterogeneity of different clinical presentation, we further did a subgroup analysis on clinical outcomes of STEMI and NSTEMI/UA patients. Not surprisingly, results showed STEMI subgroup contributed more to higher 2-year all-cause and cardiac mortality, MI, and in-stent thrombosis in the LMCA group.

There are several inherent limitations in our study. First, whether the lesion in LMCA is the culprit lesion causing ACS is unknown. We acknowledge that whether LMCA is the culprit vessel is an important factor determining the outcome of patients with ACS; thus, the findings of our study cannot be specifically extended to ACS patients with culprit LMCA lesions. Second, due to limited sample size in the LMCA group, statistical analysis is less reliable than large-sample comparison. Despite using Cox regression analysis and PSM to adjust for unmatched baseline characteristics, potential unknown risk factors still exist. Third, IVUS results, postdilatation results, whether patients received emergency or selective PCI, position of lesion in LMCA, stenting technique for bifurcation lesions, and complications including cardiogenic shock are unknown. Finally, trials with longer follow-ups are needed to further confirm our findings.

## 5. Conclusion

In patients presenting with ACS, LMCA-targeted PCI is associated with higher risk of 2-year cardiac death, MI, in-stent thrombosis, and stroke. LMCA-targeted PCI is an independent risk factor for 2-year MI. Our findings could provide useful prognosis information to clinicians and ACS patients with significant LMCA lesion.

## Figures and Tables

**Table 1:** Baseline patient characteristics.

	Before PSM			After PSM		
	LMCA	Non-LMCA	*p* value	LMCA	Non-LMCA	*p* value
	(*n* = 155)	(*n* = 6274)		(*n* = 150)	(*n* = 150)	
Age	61.91 ± 9.80	58.29 ± 10.41	**<0.001**	61.96 ± 9.68	60.97 ± 11.02	0.411

Female, *n* (%)	40 (25.8)	1452 (23.1)	0.438	39 (26.0)	41 (27.3)	0.794

Body mass index, kg/m^2^	25.58 ± 2.98	25.89 ± 3.20	0.233	25.64 ± 2.98	25.41 ± 3.58	0.545

Risk factors and history, *n* (%)						
Smoker	89 (57.4)	3755 (59.9)	0.542	86 (57.3)	79 (52.7)	0.417
Diabetes	56 (36.1)	1791 (28.5)	**0.039**	55 (36.7)	52 (34.7)	0.718
Hypertension	92 (59.4)	3993 (63.6)	0.273	89 (59.3)	95 (63.3)	0.477
Hyperlipidemia	101 (65.2)	4125 (65.7)	0.879	98 (65.3)	105 (70.0)	0.388
Prior myocardial infarction	27 (17.4)	828 (13.2)	0.126	26 (17.3)	18 (12.0)	0.192
Prior stroke	20 (12.9)	674 (10.7)	0.392	19 (12.7)	19 (12.7)	1.000

Laboratory tests						
Leukocyte, ×10^9^/L	7.22 ± 1.97	7.08 ± 2.10	0.430	7.22 ± 1.97	7.27 ± 2.21	0.828
Platelet, ×10^9^/L	209.97 ± 59.71	208.51 ± 57.02	0.757	209.97 ± 59.71	211.13 ± 58.61	0.865
Hemoglobin, g/L	134.16 ± 14.73	140.83 ± 16.15	**<0.001**	134.16 ± 14.73	134.73 ± 15.71	0.745
Creatinine, *μ*mol/L	76.55 ± 18.90	76.03 ± 16.41	0.697	76.40 ± 18.94	76.95 ± 17.96	**0.046**
GFR, ml/min	87.71 ± 16.22	91.03 ± 15.54	**0.009**	87.79 ± 16.36	88.29 ± 17.44	0.799
LVEF, %	62.82 ± 6.95	62.28 ± 7.46	0.375	62.96 ± 6.85	60.83 ± 9.48	**0.028**

Clinical presentation						
STEMI	24 (15.5)	1421 (22.6)	**0.035**	23 (15.3)	36 (24.0)	0.059
NSTEMI	11 (7.1)	464 (7.4)	0.888	10 (6.7)	12 (8.0)	0.658
UA	120 (77.4)	4389 (70.0)	**0.045**	117 (78.0)	102 (68.0)	0.051

Medication at discharge, *n* (%)						
Aspirin	154 (99.4)	6185 (98.6)	0.726	149 (99.3)	149 (99.3)	1.000
Clopidogrel	154 (99.4)	6258 (99.7)	0.340	149 (99.3)	149 (99.3)	1.000
Ticagrelor	1 (0.6)	13 (0.2)	0.290	1 (0.7)	1 (0.7)	1.000
*β*-blockers	146 (94.2)	5575 (88.9)	**0.036**	142 (94.7)	141 (94.0)	0.803
Calcium channel blockers	80 (51.6)	3121 (49.7)	0.646	76 (50.7)	68 (45.3)	0.355
Nitrates	154 (99.4)	6141 (97.9)	0.382	149 (99.3)	147 (98.0)	0.622
Statins	150 (96.8)	6011 (95.8)	0.552	146 (95.3)	140 (93.3)	0.169

Values are mean ± SD or *n* (%). GFR = glomerular filtration rate; LVEF = left ventricular ejection fraction; STEMI = ST elevation myocardial infarction; NSTEMI = non-ST elevation myocardial infarction; UA = unstable angina.

**Table 2:** Coronary angiographic findings and percutaneous interventional therapies.

	Before PSM			After PSM		
	LMCA	Non-LMCA	*p* value	LMCA	Non-LMCA	*p* value
	(*n* = 155)	(*n* = 6274)		(*n* = 150)	(*n* = 150)	
SYNTAX score						
Before procedure	20.11 ± 9.98	11.34 ± 7.83	**<0.001**	19.85 ± 9.89	19.89 ± 9.88	0.970
After procedure	2.91 ± 5.29	3.32 ± 5.67	0.368	2.80 ± 5.28	4.49 ± 6.72	**0.016**
Unprotected LMCA	147 (94.8)	—	—	142 (94.7)	—	—
Trivessel disease, %	21 (13.5)	107 (1.7)	**<0.001**	13 (8.7)	0 (0)	**<0.001**
Total occlusion, %	33 (21.3)	1350 (21.5)	0.946	32 (21.3)	64 (42.7)	**<0.001**
Puncture site, %						
Femoral artery	20 (12.9)	431 (6.9)	**0.014**	20 (13.3)	18 (12.0)	0.558
Radial artery	132 (85.2)	5749 (91.6)		127 (84.7)	131 (87.3)	
Other approaches	3 (1.9)	94 (1.5)		3 (2.0)	1 (0.7)	
Staged PCI, %	53 (34.2)	570 (9.1)	**<0.001**	53 (35.3)	55 (36.7)	0.810
IVUS usage, %	71 (45.8)	261 (4.2)	**<0.001**	71 (47.3)	71 (47.3)	1.000
IABP usage, %	18 (11.6)	81 (1.3)	**<0.001**	18 (12.0)	18 (12.0)	1.000
Successful PCI, %	152 (98.1)	6166 (98.3)	0.751	148 (98.7)	148 (98.7)	1.000
PTCA only, %	60 (38.7)	1024 (17.1)	<0.001	60 (40.0)	59 (39.3)	
Stent type						
BMS, %	0 (0)	46 (0.8)	0.285	0 (0)	1 (0.7)	1.000
DES, %						
1G-DES	17 (11.0)	1150 (19.2)	**0.019**	16 (10.7)	13 (8.7)	0.558
2G-DES	59 (38.1)	2706 (45.1)	0.208	57 (38.0)	53 (35.3)	0.632
BP-DES	19 (12.3)	957 (16.0)	0.305	23 (15.3)	17 (11.3)	0.308
Others	0 (0)	88 (1.5)	0.277	0 (0)	1 (0.7)	1.000
Blended multiple DESs	0 (0)	28 (0.5)	1.000	0 (0)	0 (0)	1.000

Values are mean ± SD or *n* (%). SYNTAX = SYNergy between percutaneous coronary intervention with TAXus and cardiac surgery; LMCA = left main coronary artery; PCI = percutaneous coronary intervention; IVUS = intravascular ultrasound; IABP = intraaortic balloon pump; PTCA = percutaneous transluminal coronary angioplasty; BMS = bare metal stent; DES = drug-eluting stent; 1G = first generation; 2G = second generation; BP = biodegradable polymer.

**Table 3:** Two-year clinical outcomes.

	Before PSM			After PSM		
	LMCA (*n* = 155)	Non-LMCA (*n* = 6274)	*p* value	LMCA (*n* = 150)	Non-LMCA (*n* = 150)	*p* value
All-cause death	4 (2.6)	82 (1.3)	0.153	3 (2.0)	2 (1.3)	1.000
Cardiac death	4 (2.6)	47 (0.7)	**0.034**	3 (2.0)	0 (0)	0.247
Myocardial infarction	11 (7.1)	116 (1.8)	**<0.001**	11 (3.0)	2 (1.3)	**0.011**
Target vessel related	8 (5.2)	52 (0.8)	**<0.001**	8 (5.3)	3 (2.0)	0.125
Target lesion related	2 (1.3)	31 (0.5)	0.188	2 (1.3)	1 (0.7)	1.000
Unplanned revascularization	10 (6.5)	546 (8.7)	0.325	10 (6.7)	15 (10.0)	0.296
Target vessel related	9 (5.8)	146 (94.2)	0.722	9 (6.0)	9 (6.0)	1.000
Target lesion related	7 (4.5)	148 (95.5)	0.755	7 (4.7)	7 (4.7)	1.000
In-stent thrombosis	7 (4.5)	51 (0.8)	**<0.001**	6 (4.0)	2 (1.3)	0.282
Stroke	6 (3.9)	88 (1.4)	**0.025**	5 (3.3)	6 (4.0)	0.759
Bleeding	11 (7.1)	401 (6.4)	0.723	11 (7.3)	6 (4.0)	0.212
MACCE	24 (15.5)	768 (12.2)	0.225	22 (14.7)	23 (15.3)	0.872

MACCE = major adverse cardiac and cerebrovascular events.

**Table 4:** 2-year clinical outcomes of STEMI and UA/NSTEMI subgroups.

	STEMI			UA/NSTEMI		
	LMCA (*n* = 24)	Non-LMCA (*n* = 1421)	*p* value	LMCA (*n* = 131)	Non-LMCA (*n* = 4853)	*p* value
All-cause death	3 (12.5)	28 (3.0)	**0.013**	1 (0.8)	54 (1.1)	1.000
Cardiac death	3 (12.5)	19 (1.3)	**0.005**	1 (0.8)	28 (0.6)	0.539
Myocardial infarction	4 (16.7)	31 (2.2)	**0.002**	7 (5.3)	85 (1.8)	**0.010**
Unplanned revascularization	1 (4.2)	129 (9.1)	0.717	9 (6.9)	417 (8.6)	0.487
In-stent thrombosis	3 (12.5)	14 (1.0)	**0.002**	4 (3.1)	37 (0.8)	**0.022**
Stroke	0 (0)	16 (1.1)	1.000	6 (4.6)	72 (1.5)	**0.016**
Bleeding	3 (12.5)	77 (5.4)	0.143	8 (6.1)	324 (6.7)	0.796
MACCE	6 (25.0)	185 (13.0)	0.118	18 (13.7)	583 (12.0)	0.549

STEMI = ST elevation myocardial infarction; NSTEMI = non-ST elevation myocardial infarction; UA = unstable angina; MACCE = major adverse cardiac and cerebrovascular events.

## Data Availability

The clinical data used to support the findings of this study are available from the corresponding author upon request.
